# Prevalence of Perinatal Central Nervous System Anomalies in East Azarbaijan-Iran

**Published:** 2011-09-25

**Authors:** M. Ghavami, R. Abedinzadeh

**Affiliations:** 1 Assistant Professor, Department of Radiology, Shohada Hospital, Tabriz University of Medical Sciences, Tabriz, Iran; 2 Day Medical Imaging Center, Tabriz, Iran

**Keywords:** Ultrasound, Prenatal, Fetal Anomaly, Central Nervous System

## Abstract

**Background/Objective:**

Central nervous system (CNS) anomalies are the most serious congenital abnormalities. Ultrasound examination is an effective and noninvasive modality for prenatal diagnosis of these anomalies. The purpose of the current study was to determine the frequency of CNS and associated abnormalities.

**Patients and Methods:**

A total of 22500 pregnant women who were referred by obstetricians/gynecologists for routine work up of pregnancy were scanned over a period of 3 years by two expert sonologists in a referral center using high resolution ultrasound unit.

**Results:**

After transabdominal sonographic examination of 22500 pregnant women, 112 (0.5%) fetuses were detected with CNS anomalies, some of whom had more than one anomaly. Forty-one (37%) Chiari malformations, 26 (23%) monro and aqueductal stenosis cases, 18 (16%) anencephaly cases, nine (8%) encephaloceles, seven (6%) microcephalies, five (4%) Dandywalker syndromes, two (2%) arachnoid cysts, two (2%) agenesis of corpus callosum cases, one (1%) holoprosencephaly and one (1%) schizencephaly were reported in our study.

**Conclusion:**

According to our results, Chiari malformation and hydrocephalus were the most prevalent anomalies of CNS congenital abnormalities in East Azarbaijan, Iran. An accurate diagnosis depends upon fetal age, amniotic fluid volume, fetal position, operator experience and careful evaluation of the associated malformations, which are often present.

## Introduction

Central nervous system congenital anomalies are the most common abnormalities of all malformations [1]. Sonographic examination is the choice modality and an effective method for diagnosis. It has been used for more than three decades as the main modality to help diagnose fetal CNS anomalies [2]. Several studies have shown an accuracy of 92% to 99.7% for ultrasonographic detection of CNS anatomic anomalies [3][4]. Routine anomaly scan during the antenatal period has become a part of obstetric care and the best time for fetal malformation scanning is approximately at 11-14, 18-20 and 28-30 weeks of gestation. The aim of this study was to estimate the incidence of major CNS anomalies in east Azarbaijan that could be detected by routine ultrasound examination.

## Patients and Methods

In the current study, 22500 pregnant women were scanned in a referral center over a period of 3 years from March 2005 to March 2008 by two expert operators with the use of high resolution ultrasound unit with 3-5 MHz transabdominal transducers. (General electric logic 500α, logic 200α). They were all referred by obstetricians/gynecologists for routine work up of pregnancy. East Azarbaijan was divided into 5 regions; namely, Tabriz (as the central part), Maragheh, Ahar, Marand and Miyaneh.

Every ultrasound scan took about 15-30 minutes. Fetuses' brains and spines were scanned in axial, coronal and sagittal sections through most important anatomic areas; i.e., transventricular, transthalamic and transcerebellar and spinal canal planes. Abnormal contours of the head and spine and empty or enlarged brain cavities were considered as important diagnostic markers for the detection of CNS anomalies in early pregnancy in the current study.

All delivered abnormal fetuses were followed by calling their mothers and asking about the child’s health. Alive and still births were followed by the obstetrician’s and pediatrician’s report.

Data were presented as mean±SD frequency and percent and inter-observer agreement was evaluated using kappa coefficient. The chi-square test and independent samples t test were used to compare the different groups. SPSS 15 for Windows (SPSS Inc., Chicago, Illinois) was used for statistical analysis.

## Results

In sonographic examination of these 22500 pregnant women, 112 (0.5%) fetuses were detected with CNS anomalies, some of whom had more than one anomaly. The mean age of mothers without fetus anomaly was 22.189.44± years and in mothers with CNS anomalies in their fetuses the mean age was 34.217.53± years. The mothers had an age range of 25-42 years. The results of the t-test for independent groups showed a statistically significant difference (P=0.02). Gross CNS anomalies observed included Chiari malformations, 41 (37%); monro and aqueductal stenosis, 26 (23%); anencephaly, 18 (16%); encephalocele, 9 (8%); microcephaly, 7 (6%); Dandywalker syndrome, 5 (4%); arachnoid cyst, 2 (2%); agenesis of corpus callosum, 2 (2%); holoprosencephaly, 1 (1%); schizencephaly, 1 (1%) (Table 1).

**Table 1:** Frequency of CNS Anomalies

**Type of Anomaly**	**Number of Fetuses**	**Percentage**
Chiari Malformation	41	36.6%
Monro and Aqueductal Stenosis	26	33.2%
Anencephaly	18	16%
Encephalocele	9	8%
Microcephaly	7	5.3%
Dandy Walker Syndrome	5	3.5%
Agenesis of Corpus Callosum	2	1.7%
Arachnoid Cyst	2	1.7%
Holoprosencephaly	1	0.89%
Schizencephaly	1	0.89%
Total	112	100%

Among the assessed fetuses, 58 (52%) were male and 54 (48%) were female. The mean age in mothers with male fetuses was 33.526.91± years and 34.947.21± years for mothers with female fetuses and no significant difference was detected (P=0.32).

Associated anomalies detected included clubfoot, 14; polycystic kidney, 6; polyhydramnios, 5; Meckel Gruber syndrome, 3; cleft lip and palate, 2; cardiomegaly, 1; umbilical hernia, 1; microphtalmia, 1 (Table 2).

**Table 2:** Associated Anomalies

**Type of Anomaly**	**Number of Fetuses **	** Percentage**
Club Foot	14	42.4%
Polycystic Kidney	6	18.1%
Polyhydramnios	5	15.1%
Meckel Gruber Syndrome	3	9%
Cleft Lip and Palate	2	6%
Cardiomegaly	1	3%
Umbilical Hernia	1	3%
Microphthalmia	1	3%
Total	33	100%

Geographical distribution of CNS anomalies has been demonstrated in Table 3.

**Table 3:** Distribution of CNS Anomalies in Different Regions of East Azarbaijan Province

Tabriz	68 (60.7%)
Maragheh	14 (12.5%)
Ahar	10 (8.9%)
Marand	12 (10.7%)
Miyaneh	8 (7.11%)

Inter-observer agreement was confirmed by kappa coefficient (kappa=0.79, P<0.001)

## Discussion

The incidence of CNS anomalies varies in developed and non-developed countries due to folic acid supplementation [2]. Additionally, it can follow a different pattern in one country based on regional food and nutritional habits. For instance, in our study, the overall incidence of major CNS anomalies was 5 per 1000 (0.5%). In one study from Khuzestan-Iran, the incidence of CNS anomalies was reported as 40 per 3012 (1.32%) pregnant women [3]. In another population based study by Monteagudo, 55226 pregnancies were evaluated and 143 (0.26%) infants with CNS defects were found [4]. While in a study published by Balakumar et al. on 857 pregnant cases, 336 (39.21%) abnormal fetuses with CNS malformations were reported [5].

The most serious complications of major CNS anomalies are disability and becoming bedridden. Thus, early diagnosis and therapeutic abortion of these major CNS anomalies is of great help to the health economy [1]. Whereas, care of the affected person costs about 250,000 US dollars per person per lifetime in the United States of America [2].

With the widespread use of prenatal ultrasonography, fetal CNS anomalies are being detected increasingly [1]. Since three dimensional and Doppler ultrasound scan have not clearly demonstrated their superiority over the routine two dimensional ultrasound imaging, prenatal ultrasonography has been based on two dimensional techniques. Color and power Doppler ultrasound scan may be used mainly to identify umbilical cord and cerebral vessels and 3D ultrasound can help to detect the lesions with complex anatomy [1]. Sonographic examination is an accurate and non-invasive non-ionizing and relatively inexpensive diagnostic test for determining prenatal anomalies [4]. The first trimester ultrasound examination can detect the majority of anencephalies [6].

Structures that are usually noted on a basic ultrasound examination of the fetal CNS include head shape, lateral ventricles, cavum septum pellucidum, thalami, cerebellum, cisterna magna and the spine. Open neural tube defects are the most common CNS malformations. Anencephaly, the single common open neural tube defect, is so severe that it is immediately detected when attempting to perform biometry of the fetal head.

Gestational age is a very important parameter for normal ultrasound appearance of the CNS system [2]. For example, the corpus callosum can not be detected at 14 weeks of gestation because these structures appear after 18-20 weeks of gestation [5]. On the other hand, the other technical factors that may cause inadequate ultrasound images are poor fetal position, obesity and oligohydramnios.

Neural tube defects are associated with an elevated maternal serum α-fetoprotein (MSAFP). In the past, all women with elevated MSAFP were offered amniocentesis as the diagnostic test [7], since elevated levels of amniotic fluid AFP together with elevated acetyl cholinesterase are considered to be diagnostic for fetal NTD (neural tube defect). Currently a detailed ultrasound scan is indicated in women with an elevated MSAFP. The sensitivity for the prenatal diagnosis of NTDs using ultrasound in experienced hands is excellent [8]. As an example, the sensitivity for anencephaly has been reported to be 100 percent and the overall sensitivity for the detection of NTD is 97 percent with a 100 percent specificity [6].

Several studies have shown that routine ultrasound is more effective than MSAFP alone in identifying fetuses with NTD. For instance, in one study, the routine second trimester ultrasound detected 96% of the NTDs compared to 78% by MSAFP alone [6]. According to our experience, the best time for fetal CNS malformations is about 11-14, 18-20 and 28-30 weeks of gestation.
